# Building Social Cohesion Through Intergroup Contact: Evaluation of a Large-Scale Intervention to Improve Intergroup Relations Among Adolescents

**DOI:** 10.1007/s10964-021-01400-8

**Published:** 2021-02-18

**Authors:** Nils Karl Reimer, Angelika Love, Ralf Wölfer, Miles Hewstone

**Affiliations:** 1 University of Oxford, Oxford, UK; 2 Deutsches Zentrum für Integrations- und Migrationsforschung, Berlin, Germany

**Keywords:** Intergroup contact, Intergroup relations, Interventions, Adolescents, Youth engagement programs

## Abstract

Past research has found intergroup contact to be a promising intervention to reduce prejudice and has identified adolescence as the developmental period during which intergroup contact is most effective. Few studies, however, have tested whether contact-based interventions can be scaled up to improve intergroup relations at a large scale. The present research evaluated *whether* and *when* the National Citizen Service, a large-scale contact-based intervention reaching one in six 15- to 17-year-olds in England and Northern Ireland, builds social cohesion among adolescents from different ethnic backgrounds. In a diverse sample of adolescents (*N* = 2099; *M*_age_ = 16.37, age range: 15–17 years; 58% female), this study used a pretest–posttest design with a double pretest to assess the intervention’s effectiveness. Controlling for test–retest effects, this study found evidence that the intervention decreased intergroup anxiety and increased outgroup perspective-taking—but not that it affected intergroup attitudes, intergroup trust, or perceptions of relative (dis-)advantage. These (small) effects were greater for adolescents who had experienced less positive contact before participating and who talked more about group differences while participating. These findings suggest that the intervention might not immediately improve intergroup relations—but that it has the potential to prepare adolescents, especially those with less positive contact experiences before the intervention, for more positive intergroup interactions in the future.

## Introduction

Social psychologists not only seek to understand intergroup relations, but also to apply what they have learnt to foster more cohesive and equal societies. From the outset, research on intergroup contact has focused on its potential to reduce prejudice and improve intergroup relations (Paluck et al., 2019). Recent meta-analyses, reviewed below, have synthesized the available evidence for the effectiveness of intergroup contact in real-life settings. All but one study (Al Ramiah and Hewstone, 2012) in these meta-analyses, however, examined isolated, small-scale interventions, which thus could not provide evidence about the potential of contact-based interventions to improve intergroup relations at scale. To provide this evidence, the present research evaluated whether participating in a large-scale contact-based intervention improves a range of outcomes relevant for interethnic relations and social integration. Unlike most published studies, this study examined an intervention that reaches a significant proportion of the relevant population (one in six 15- to 17-year-olds in England and Northern Ireland), is intended to facilitate positive intergroup contact experiences, and recurs annually. This study therefore provides a rare test of the viability of large-scale applications of intergroup contact research in real-life settings. This intervention targets adolescents who are in the age group in which intergroup attitudes are developing before they crystallize in early adulthood and who are thus particularly receptive to the positive effects of intergroup contact experiences (Wölfer et al., 2016).

### Contact-Based Interventions

Numerous cross-sectional, longitudinal, and experimental studies provide evidence that intergroup contact can improve intergroup relations (for a meta-analysis, see Pettigrew and Tropp, 2006). This evidence makes contact-based interventions one of the most promising avenues for reducing prejudice (Paluck and Green, 2009). Recent meta-analyses have reviewed the evidence for the effectiveness of intergroup contact for improving intergroup relations in real-life settings. Beelmann and Heinemann (2014) examined interventions to reduce prejudice among children and adolescents, and found that interventions based on direct contact experiences were among the most effective. Lemmer and Wagner (2015) found that contact-based interventions were indeed effective at reducing ethnic prejudice. Paluck et al. (2019) considered only the most rigorously conducted studies that involved random assignment and delayed outcome measures and found evidence for positive but heterogeneous effects of contact-based interventions on outgroup attitudes. Few of the studies reviewed in these meta-analyses, however, evaluated interventions with more than a few hundred participants. As such, these meta-analyses reveal a lack of evidence for the effectiveness of *large-scale* contact-based interventions, which limits the extent to which policy makers can harness intergroup contact to improve intergroup relations in real-life settings.

An exception is a recent evaluation of a recurring, large-scale intervention (the Malaysian National Service program) which facilitated direct intergroup contact between members of different ethnic groups and reached one in four Malaysian 18-year-olds (Al Ramiah and Hewstone 2012). This study found limited evidence for a positive effect of program participation on interethnic relations but cautioned that the intervention was not designed to facilitate positive intergroup contact experiences and may not have met the conditions necessary for intergroup contact to be effective. Therefore, there is still a lack of evidence about whether interventions designed on the basis of intergroup contact theory can effectively improve intergroup relations for a significant proportion of a population. The present research seeks to address this question by evaluating an intervention designed to facilitate positive intergroup contact experiences and reaching thousands of adolescent participants every year.

Developmental science suggests that intergroup contact improves intergroup relations most effectively when experienced at a younger age. For example, the ‘impressionable years hypothesis’ (Krosnick and Alwin, 1989) suggests that attitudes develop at a young age before they tend to stabilize in adulthood. This is driven, in part, by specific cognitive and psychosocial dynamics that seem to ‘softwire’ adolescents for intergroup contact experiences and thereby shape their intergroup relations in life. Specifically, during adolescence (a) the understanding of group norms increases and the perceived salience of social norms and thus the potential for intergroup bias peaks (Rutland et al., 2010), (b) peer relationships, which socialize (intergroup) attitudes and behaviors, reach maximum importance (Brechwald and Prinstein, 2011), and (c) an identity—including an ethnic identity (Spiegler et al., 2019)—forms (Erikson, 1963). Further highlighting the significance of intergroup contact during adolescence, recent longitudinal research among a large sample of Swedish individuals (aged 13 to 26 years) indicated that intergroup contact during adolescence is particularly relevant, perhaps even necessary, for the development of favorable intergroup attitudes in adulthood (Wölfer et al., 2016; Study 2). Diverse school environments provide adolescents with opportunities for experiencing intergroup contact (Birtel et al., 2020) but are not available to many adolescents. The present research examines a contact-based intervention targeting 15- to 17-year-olds to evaluate whether it can facilitate the development of positive intergroup relations among adolescents.

### Moderators of Intergroup Contact

Meta-analyses revealed substantial heterogeneity in the effects of contact-based interventions (Lemmer and Wagner, 2015; Paluck et al., 2019). Studies of potential moderators can help explain this heterogeneity by examining who is most likely to benefit from participating in contact-based initiatives and what form an intervention needs to take to be most effective.

#### Contact before the intervention

Before an intervention, participants will have had a range of contact experiences with outgroup members. Like many psychological effects, the effects of intergroup contact are likely subject to the law of diminishing returns. As such, it can be expected that a contact-based intervention will be more effective for participants with less prior positive contact. In line with this hypothesis, participating in a contact-based intervention was associated with a greater improvement of outgroup attitudes for participants with fewer positive prior contact experiences (Laurence, 2020). Conversely, it can be expected that a contact-based intervention will be more effective for participants with more prior negative contact. In their research on imagined contact, Birtel and Crisp (2012) argued that negative contact could make subsequent positive contact more effective. Árnadóttir et al. (2018) reviewed the various ways that negative contact and positive contact could interact (see also Fell, 2015) and found cross-sectional evidence for a stronger relationship between positive contact and more favorable outgroup attitudes among participants who also reported more negative contact. However, prior negative contact did not moderate the effectiveness of a contact-based intervention (Laurence, 2020). The present research tests whether this finding can be replicated and examines the potential moderating effect of participants’ positive and negative contact experiences before the intervention.

#### Contact during the intervention

During an intervention, participants will experience a range of different types of contact with outgroup members. It can be expected that a contact-based intervention will be more effective for participants who experience high-quality contact during the intervention. Meta-analytic evidence (Pettigrew and Tropp, 2006) suggested that the prejudice-reducing effects of intergroup contact are not limited to what Allport (1954) described as the “optimal” conditions for contact (equal status, cooperation, common goals, institutional support), but that they emerge for all broadly positive contact experiences. Recent studies have shown that, whereas positive contact reduces prejudice, negative contact might increase negative outgroup attitudes (e.g., Hayward et al., 2017). These findings support the argument that contact-based interventions should carefully consider not just how much intergroup contact they facilitate, but also how participants experience the quality of these interactions.

Researchers have debated whether intergroup contact should be structured to emphasize commonalities or differences between groups in order to most effectively improve intergroup relations. Brewer and Miller (1984) argued that contact improves intergroup relations most effectively when it emphasizes similarities, deemphasizes group memberships—and thus facilitates ‘interpersonal’ interactions. Hewstone and Brown (1986) instead argued that, for the beneficial effects of intergroup contact to generalize to all outgroup members, group memberships need to be salient and outgroup members need to be perceived as typical during contact—thus facilitating “intergroup” interactions. Integrating these perspectives, Brown and Hewstone (2005) argued that the most effective contact situations would be those that facilitate both “interpersonal” and “intergroup” interactions. This argument was supported by a study finding that participants reacted more positively to imagining contact situations that featured both similarities and differences than to situations that emphasized only similarities or only differences between groups (Ioannou et al., 2017). The present research thus considers to what extent the effectiveness of program participation depends on participants’ intergroup contact experiences before and during a large-scale contact-based intervention.

### National Citizen Service

The National Citizen Service (NCS) is a recurring youth-engagement program in England and Northern Ireland aiming to bring together young people from different backgrounds to help bring about “a more responsible, cohesive and engaged society” (National Audit Office, 2017, p. 6). It aligns itself closely with intergroup contact research, seeking to “help young people to build trusting and meaningful relationships with those from other backgrounds” by supporting their ability to connect, understand, empathize, and work well with outgroup members (The Challenge, 2019, p. 4). Unlike most contact-based interventions (Lemmer and Wagner 2015) the NCS takes place outside of formal education and is delivered by several providers across England and Northern Ireland. Instead of reproducing the relative demographic homogeneity of many educational settings (see Johnston et al., 2006), the NCS deliberately brings together adolescents from the same region in small, diverse teams broadly reflecting the demographic composition of the youth population within their Local Authority. Over 3–4 weeks, participants work collaboratively in team- and skill-building activities (National Audit Office, 2017). The program, which is delivered by several organizations, has seen significant investment from the UK Government, with £634 million (95%) of governmental spending on youth services between 2014 and 2018 being allocated to the NCS (Walker, 2018). In 2018, 99,674 eligible 15- to 17-year-olds participated in the NCS, that is, one in six of the target population (National Citizen Service Trust, 2020).

Despite its scope and financing, empirical evidence about the NCS’s effect on intergroup relations remains scarce. Laurence (2019, 2020) published the only other rigorous empirical evaluation to date. Using a quasi-experimental pretest–posttest design with a matched control group, he found that participating in the NCS was associated with small, significant increases in interethnic contact (2019) and favorable outgroup attitudes (2020) 4–6 months later. Laurence (2020) found some evidence that the NCS improved outgroup attitudes, in part, by increasing how often adolescents had positive contact experiences *after* the intervention, and that the NCS had a greater effect on adolescents with less positive contact *prior* to the intervention. The present research seeks to replicate this study (using near-identical measures of intergroup attitudes and contact) but also to extend it in three main ways.

First, Laurence (2020) considered only outgroup attitudes as a relevant outcome while the current study examines a broader range of direct and indirect effects of intergroup contact on intergroup relations. Most studies of intergroup contact have examined whether it can improve outgroup attitudes, that is, foster more positive evaluations of the outgroup and its members (Pettigrew and Tropp, 2006). Some studies have found intergroup contact to also build outgroup-directed trust (Tam et al., 2009) which, unlike attitudes, entails making benign assumptions about other people’s behavior (Molm et al., 2000) and is thus crucial to positive intergroup relations. The current study considers both outgroup attitudes and outgroup trust as potential *direct* effects of the contact-based intervention. Reducing intergroup anxiety (that is, feelings of unease and insecurity about interacting with outgroup members; Stephan and Stephan 1985) and increasing outgroup perspective-taking (that is, the ability to see a situation from the outgroup’s point of view; Aberson and Haag 2007) are the most important processes by which intergroup contact reduces prejudice (Pettigrew and Tropp, 2008). In addition, reduced intergroup anxiety is associated with more positive expectancies about future contact (Gómez et al., 2011) and less contact avoidance (Kenworthy et al., 2016) and can thus, alongside outgroup perspective-taking, perpetuate further positive intergroup experiences over time. The current study considers both intergroup anxiety and outgroup perspective-taking as potential *indirect* effects of the contact-based intervention with the potential to facilitate more positive intergroup relations over time.^1^

Second, Laurence (2020) examined only *prior* positive and negative contact as moderators of the intervention’s effectiveness and did not include measures of contact *during* the intervention. This is crucial if one seeks to attribute any success of the intervention to its facilitation of, and participants’ participation in, intergroup contact—and if one wants research to inform the design of future interventions. The current study included measures of both the perceived *quality* (e.g., how cooperative or competitive it was) and *content* (e.g., whether it emphasized intergroup commonalities or differences) of contact during the intervention, making it possible to assess how contact *during* the intervention needs to be structured for optimal effectiveness.

Third, Laurence (2020) did not differentiate between specific outgroups in his measures and analyses. In contrast, the current study examined how the NCS affects relations between two ethnic minority and one ethnic majority group. This is crucial because past research has shown intergroup contact to be less effective for minority-group members (Tropp and Pettigrew, 2005).

Relatedly, the present research acknowledges that contact could, in principle, have unintended negative consequences for members of minority groups by clouding their perceptions of the disadvantage and discrimination they suffer (Dixon et al., 2012). This, in turn, might entrench social inequality because perceived discrimination could otherwise motivate disadvantaged-group members to engage in collective action for social change (van Zomeren et al., 2008). In line with this argument, cross-sectional (Dixon et al., 2010), longitudinal (Tropp et al., 2012), and experimental (Saguy et al., 2009) evidence suggests that contact with an advantaged group is associated with lower levels of perceived discrimination among members of disadvantaged groups—though other research found that these effects are confounded by negative contact experiences (Reimer et al., 2017) and do not replicate among disadvantaged groups in some settings (Reimer et al. 2020). Conversely, contact could be effective to the extent that it, respectively, heightens majority members’ awareness of their own group’s advantage and the minority’s disadvantage—and thus make them allies in the struggle for social justice (Craig et al. 2020). The current study therefore included measures of perceived relative (dis-)advantage.

## Current Study

The current study evaluated to what extent participating in the National Citizen Service (NCS) improves interethnic relations and prepares adolescents of diverse ethnic groups (Asian, Black, White) for future intergroup contact. As such, it addresses the need for research on the effectiveness of large-scale contact-based interventions in adolescence, the developmental period in which intergroup contact is thought to be the most effective.

In line with the aims of the intervention and informed by intergroup contact theory, this study, first, tested the hypothesis that participating in the NCS would be associated with more favorable intergroup attitudes and more intergroup trust—and thus directly improve intergroup relations. Second, this study tested the hypothesis that participating in the NCS would be associated with less intergroup anxiety and more outgroup perspective-taking—and thus prepare participants for future intergroup contact experiences and indirectly improve intergroup relations. Third, this study tested the hypothesis that participating in the NCS would be associated, for Asian and Black minority-group participants, with feeling less disadvantaged relative to White people after the intervention and, for White majority-group participants, with feeling more advantaged relative to Asian and Black people.

The current study not only considered *whether* but also *for whom* participating in the NCS improved intergroup relations. Drawing on research on moderators of contact effects, this study contributed further insights into “what specific aspects of the contact are reducing participants’ prejudice” (Paluck et al. 2019, p. 25). Specifically, this study explored to what extent the effect of participating in the intervention varied as a function of participants’ contact experiences during the intervention (contact quality, contact content) and their contact experiences before the intervention (positive/negative contact). Without clear directional predictions for some moderator variables, these analyses should be considered exploratory.

## Method

### Design

This evaluation was conducted in collaboration with an organization who, when this study was conducted, organized the NCS for around 45,000 adolescents across England. Admission to the program was administered centrally and was voluntary. As such, it was not possible to use a study design with random assignment and a control group. Instead, this study used a pretest–posttest design with a double pretest (Bell, 2010) in which participants completed surveys two weeks before, immediately before, and immediately after the 3–4 week-long intervention. This study design made it possible to control for test–retest effects (such as maturation or regression to the mean) that threaten the internal validity of the simple pretest–posttest design. We report how we determined our sample size, all data exclusions, all manipulations, and all measures in the study.

### Participants

In 2017, 45,000 adolescents participated in the NCS through the organization. Of these, 2,099 (*M*_age_ = 16.37, age range: 15–17 years; 1224 female, 875 male) were recruited from 20 locations that were selected to be broadly representative of the different regions of England and that included both rural and urban areas. As outlined below, almost all participants in each location and cohort participated in the study (see Procedure). The sample size was determined to maximize statistical power while not placing too much of a burden on the partner organization. Of this sample, 579 (28%) were Asian,^2^ 317 (15%) were Black, 945 (45%) were White, and 258 (12%) either had a multiethnic background (*n* = 173), another ethnic background (*n* = 51), or did not provide any information on their ethnicity (*n* = 34). As such, this sample was more diverse than the population of 15- to 17-year-olds in England and Wales as a whole (8% Asian, 4% Black, 82% White; Office for National Statistics. 2018), reflecting the greater diversity of the regions for which the organization administered the program. Of all participants, 520 (25%) received free school meals, which means that they or their parents received welfare benefits. As measures referred to Asian, Black, and White people as target groups, this study included only Asian, Black, and White participants (*N* = 1,841, 88%) in the analyses. This was necessary in order to divide the target groups referred to in the measures into “ingroups” and “outgroups”.

### Procedure

Participants from 35 cohorts (35 ≤ *n* ≤ 72) at 20 locations (45 ≤ *n* ≤ 196) took part in the study. Participants were asked to complete three surveys. Participants received an invitation to participate in the first survey via an email sent two weeks before the start of the program (time 1). Of all 2,099 participants who went on to fill in at least one survey, 401 (19%) followed a link in the email to complete the online survey at time 1 with all outcome measures described below. Participants filled in the second survey after arriving at the location, but before the start of the program (time 2). Of all participants, 2,068 (99%) filled in this pen-and-paper survey with all outcome measures and the measures of positive/negative contact prior to the program, described below. Participants completed the third survey at the end of the program (time 3), that is, three to four weeks later. Of all participants, 2052 (98%) filled in this pen-and-paper survey, which included all outcome measures and the contact quality and group salience measures described below. In total, 389 (19%) participants completed all three surveys, 1632 (78%) completed the second and third surveys, and 78 (3%) completed only one (the second or third) or two (only the first and second or first and third) surveys. As reported in the supplemental materials, there was no evidence that participants who joined for the first survey differed from participants who joined for the second and third survey. Participants completed measures in the same order in each survey (perceived (dis-)advantage, outgroup perspective-taking, moderator variables, intergroup anxiety, intergroup trust, intergroup attitudes). Demographic information (see Participants) was provided, together with the (anonymized) survey responses, by the partner organization.

### Measures

For all measures, this subsection reports Spearman’s rank correlation coefficient (*r*_12_, *r*_23_) as an index of test-retest reliability and two indices of internal consistency reliability, McDonald’s omega (*ω*_1_, *ω*_2_, *ω*_3_) for multi-item measures (Dunn et al. 2014) and the Spearman-Brown statistic (*ρ*) for two-item measures (Eisinga et al. 2013).^3^ All measures were adapted from the intergroup contact literature in cooperation with the partner organization who pretested all measures in focus groups of adolescents. In addition to the ones used in the current study, participants responded to other items that were of interest to the partner organization but not relevant to the current study (for a complete list, see https://osf.io/jn725/).

#### Intergroup attitudes

This outcome was measured with an eleven-point response scale (based on Converse and Presser, 1986): “Imagine a thermometer and indicate how warm or cold you feel toward people from the following groups. If you feel warm/more favorably toward a group, choose a higher number (50–100). If you feel cold/less favorably you choose a lower number (0–50).” (0 = *cold*, 100 = *warm*; *r*_12_ = 0.48, *r*_23_ = 0.50).

#### Intergroup trust

This outcome was measured with a four-point response scale (adapted from Kenworthy et al., 2016): “Imagine a situation in which you have to rely on someone (such as leaving your bag with them for a few minutes, or working with them on a shared project). Think about a person from each of the following groups. How likely are they to take advantage of the situation, and how likely are they to be fair?” (1 = *definitely try to take advantage*, 2 = *most likely try to take advantage*, 3 = *most likely try to be fair*, 4 = *definitely try to be fair*; *r*_12_ = 0.32, *r*_23_ = 0.30).

#### Intergroup anxiety

This outcome was measured with the same three items for all participants (adapted from Stephan and Stephan 1985). Participants read the following vignette: “Imagine you are in a new school class where you do not know anyone. Everyone is from the same ethnic background, but it is different to your own ethnic background. How would you feel interacting with your new classmates?”. Participants rated three semantic-differential items with a five-point response scale: nervous–relaxed, awkward–at ease, and timid–confident (*r*_12_ = 0.48, *r*_23_ = 0.37; *ω*_1_ = 0.87, *ω*_2_ = 0.92, *ω*_3_ = 0.94). Items were reverse coded so that higher scores reflected more intergroup anxiety and a decrease over time reflected change in the hypothesized direction.

#### Outgroup perspective-taking

This outcome was measured with one item (based on Aberson and Haag, 2007) for each of two outgroups with a seven-point response scale: “How hard or easy do you find it to imagine what it would be like to grow up as a person from the following groups today?” (1 = *very hard*, 7 = *very easy*; *r*_12_ = 0.48, *r*_23_ = 0.37). Higher scores thus reflected more outgroup perspective-taking, while an increase over time reflected change in the hypothesized direction.

#### Perceived (dis-)advantage

This outcome was measured with a seven-point response scale (based on Reimer et al. 2020): “People face different obstacles and enjoy different advantages in life. On average, how hard or easy do you think it is for people from the following groups to become successful in Britain today?” (1 = *very hard*, 7 = *very easy*; *r*_12_ = 0.54, *r*_23_ = 0.50).

Measures of intergroup attitudes, intergroup trust, perceived (dis-)advantage included one item for each of two outgroups (“Asian people in Britain”, “Black people in Britain”, and/or “White people in Britain”) and for the participant’s ingroup (“People from your own ethnic background”)—that is, for three target groups per participant. For these outcome variables, participants’ ratings were transformed into two intergroup bias scores (Hewstone et al., 2002) per outcome variable by subtracting the participants’ rating of their ingroup (e.g., White) from their rating of each outgroup (e.g., Asian, Black). For intergroup attitudes and intergroup trust, negative scores thus reflected an ingroup bias, and an increase over time in either variable reflected change in the hypothesized direction. For perceived (dis-)advantage, positive scores showed that participants saw themselves at a disadvantage relative to an outgroup while negative scores showed that participants saw themselves at an advantage relative to an outgroup.

#### Contact before the intervention

Positive and negative contact before the intervention were measured with two items per outgroup, each with a five-point response scale (based on Barlow et al., 2012): “In the past year, how often have you had positive/good [negative/bad] experiences with people from the following groups?” (1 = *never*, 5 = *very often*; *r*_Asian_ = −0.03, *r*_Black_ = −0.10, *r*_White_ = −0.28).

#### Contact during the intervention

Prior to completing items on contact experiences during the intervention, participants read the following explanation (based on Islam and Hewstone 1993): “In this section, we are interested in the interactions you had with young people who are from different ethnic backgrounds than your own [during the intervention]. To what extent did you experience these interactions as shallow or intimate, and competitive or cooperative?”. Contact quality during the intervention was measured with two semantic-differential items with a seven-point response scale: shallow–intimate, competitive–cooperative (1–7; *ρ* = 0.80). The two items were aggregated into an index of contact quality. Contact content during the intervention was measured with two items, using a five-point response scale: “In these interactions, how often did you talk about the common things [the differences] in your lives and experiences?”, (1 = *never*, 5 = *very* often; *ρ* = 0.79). These items were aggregated into two indices: The *sum* of the two items reflected how often participants talked about both commonalities and differences in their lives and experiences. The *difference* between the two items reflected how much more often participants talked about differences than about commonalities in contact experiences during the intervention.

### Analysis Strategy

This study resulted in a dataset that has ordinal outcome variables (that is, items with ordered categorical response options), is longitudinal (observations nested in participants), and is hierarchical (participants nested in cohorts/locations). This dataset was analyzed using multilevel ordinal regression models predicting participants’ responses to the five outcome variables across the three time points.

These complex models were estimated in RStan (Stan Development Team, 2019) using Bayesian statistical methods. Bayesian inference involves choosing a likelihood function and prior distributions. A likelihood function links the observed data to one or more model parameters (e.g., regression coefficients) and states how likely the observed data are given different values of said model parameters. The models used in this study derived the likelihood of the observed responses from a generalized linear model with a cumulative logit link function. Prior distributions state how plausible different values of said model parameters are before considering the observed data. The models used in this study assigned conservative, weakly informative prior distributions to all model parameters.^4^ Bayesian inference applies Bayes’ theorem to update prior distributions in light of the observed data to produce posterior distributions. Other than *p* values and confidence intervals, the resulting posterior distributions have a straightforward interpretation as stating how plausible different values of the model parameters are given the observed data. The “Results” section reports point estimates, based on the median of the posterior distribution, and uncertainty intervals, enclosing the 95% most plausible values.

**Fig. 1:**
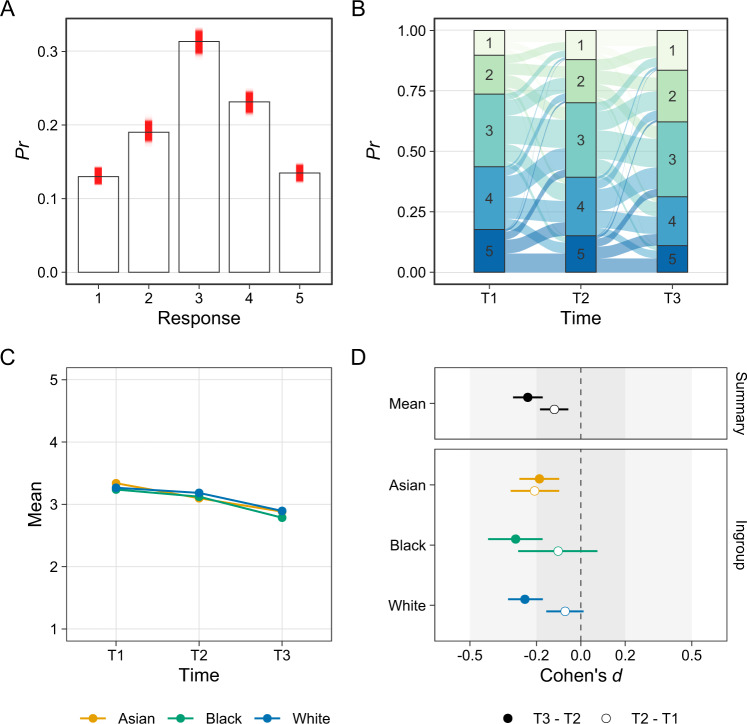
Model predictions for intergroup anxiety. **A** Proportion of each response option as observed in the data (bars) and as estimated by the ordinal regression model (red intervals). Comparing the two shows that the model fits well. **B** Estimated proportion of each response option at each time point and estimated change across time points. **C** Estimated mean score for each participant ingroup at each time point. **D** Estimated mean change from T1 to T2 and from T2 to T3 as Cohen’s *d* effect size. Effect sizes below zero indicate change in the hypothesized direction. **A** and **B** include varying (random) effects while **C** and **D** consider only fixed effects

Analyzing ordinal outcomes as if they were metric data—as is common practice in psychological research—risks distorting estimates of effect sizes and inflating rates of false-positive and false-negative findings (Liddell and Kruschke, 2018). Instead, the present analysis used cumulative ordinal regression models which estimated how likely it was that participants would choose each of the available response options (for an introduction, see Bürkner and Vuorre 2019). Figure 1A, B, for example, show posterior predictions from an ordinal regression model for participants’ intergroup anxiety ratings. As Fig. 1A shows, the model predicted that participants would choose each of five response options with the proportions Pr(*y* = 1) = 0.13, [0.13, 0.14]; Pr(*y* = 2) = 0.19, [0.18, 0.20]; Pr(*y* = 3) = 0.31, [0.30, 0.32]; Pr(*y* = 4) = 0.23, [0.22, 0.24], and Pr(*y* = 5) = 0.13, [0.13, 0.14] across time points. Comparing predicted and observed proportions shows that the model fit well. As Fig. 1B shows, the model estimated change over time and thus predicted how many participants would change their response from one option to another. From estimated proportions, one can derive the estimated means for each ingroup and time point (Fig. 1C)^5^ which, in turn, makes it possible to calculate Cohen’s *d* effect size by dividing the difference between two estimated means by the pooled standard deviation across comparison groups (Fig. 1D).

All models estimated participants’ responses as a function of an intercept (estimating participants’ responses at the start of the intervention) and the change from time 1 to time 2 (estimating change *before* the intervention), and from time 2 to time 3 (estimating change *during* the intervention). If change during the intervention was greater than change before the intervention, this difference is attributed to the intervention showing that the intervention had an effect that could not be reduced to test–retest effects. The “Results” section reports the estimated change before and during the intervention as both raw mean differences (*ΔM*_12_, *ΔM*_23_) and Cohen’s *d* effect size (*d*_12_, *d*_23_). In addition, the “Results” section reports the estimated difference between the two change scores (*d*_23_–*d*_12_) and the posterior probability that the estimated change during the intervention was greater than the estimated change before the intervention (Pr(*d*_23_ > *d*_12_)). As noted in the “Discussion” section, the lower response rate in the first survey limits the precision of these estimated differences.

The current study included participants from three ingroups (Asian, Black, White) and measured outcome variables in reference to either people from a different ethnic background in general (intergroup anxiety), to two specified outgroups (outgroup perspective-taking), or to two specified outgroups and the participant’s ingroup (intergroup attitudes, intergroup trust, perceived (dis-)advantage). Accordingly, three kinds of models were used to account for the different combinations of participant ingroups and target groups. For intergroup anxiety, the model estimated distinct effects for each of the three ingroups. For outgroup perspective-taking, the model estimated distinct effects for six combinations of participant ingroups and target outgroups. For all other outcomes, the models estimated distinct effects across all nine combinations of participant ingroups and target groups. As outlined above, model predictions were transformed for these outcomes so that effect size estimates reflected changes in intergroup bias. For example, while models estimated White participants’ attitudes toward Asian, Black, and White people, the “Results” section reports transformed estimates to reflect changes in intergroup bias against Asian and Black people (by subtracting estimates of ingroup ratings from estimates of outgroup ratings, see “Measures”).

For all outcomes (except intergroup anxiety for which there were only three relevant comparisons), the models adjusted estimates for multiple comparison by using partial pooling (Gelman et al. 2012), that is, by estimating intercepts and change over time as varying (random) effects across all relevant combinations of participant ingroups and target groups. In addition, the models estimated varying intercepts across participants to control for individual differences immediately before the intervention (time 2) and varying effects across cohorts/locations to estimate variance in change during the intervention (from time 2 to time 3).^6^

The current study included five moderator variables (positive and negative contact before the intervention; contact quality during the intervention; how often participants talked about both commonalities and differences; and how much more often participants talked about differences than commonalities). A second set of models, two for each outcome variable, tested for moderation: One model estimated how these effects varied as a function of self-reported contact b*efore* the intervention (positive, negative). Another model estimated how the change before and during the intervention, as well as participants’ responses at the start of the intervention, varied as a function of the self-reported contact quality and content *during* the intervention. Again, these effects were estimated as varying across all relevant combinations of participant ingroups and target groups.

Ordinal regression is a form of logistic regression and, as such, the effects of predictor variables on the outcome variable are non-linear. When models include multiple predictor variables, as in the moderator analyses, the effect of one predictor variable depends on the levels of all other predictor variables—even when no product interaction term is included. As such, one has to consider the moderating effects of all variables in combination. To do so, the difference between change before and during the intervention (*d*_23_–*d*_12_) was derived for all combinations of participant ingroups, target groups, and levels of the five moderator variables. Figures 3 and 4 report effect-size estimates for each of those combinations. In addition, the text reports how much bigger or smaller the effect of the intervention on each outcome would have been if—all other variables held equal—all participants had reported higher (+1*SD*) or lower (−1*SD*) values on a moderator variable.

## Results

### Descriptive Statistics

Table 1 shows correlations between outcome and moderator measures, as well as means and standard deviations. Before the intervention, participants reported some ingroup bias in their intergroup attitudes (*M* = −0.41, *SD* = 1.33) but not in their intergroup trust ratings (*M* = 0.01, *SD* = 0.42) as well as a moderate amount of outgroup perspective-taking (*M* = 4.14, *SD* = 1.43, 1–7). Asian (*M* = 1.00, *SD* = 1.42) and Black (*M* = 2.36, *SD* = 1.84) participants perceived themselves to be at a *disadvantage* relative to White people whereas White participants perceived themselves to have an *advantage* relative to Asian (*M* = −0.71, *SD* = 1.19) and Black (*M* = −0.91, *SD* = 1.30) people. Participants reported to have had more positive (*M* = 3.96, *SD* = 0.93) than negative (*M* = 2.08, *SD* = 0.89) contact before the intervention. During the interventions, participants reported, on average, having some amount of high-quality contact (*M* = 4.94, *SD* = 1.18, 1–7) and talking somewhat less often about differences than about commonalities in intergroup interactions (*M* = −0.18, *SD* = 0.79).

**Table 1:** Correlations between all outcome and moderator measures, as well as means and standard deviations, for each survey

				*r*			
#	Measure	*M*	*SD*	1	2	3	4
**A** First Survey							
1	Intergroup anxiety	3.35	1.08	−0.10	−0.03	0.01	−0.05
2	Intergroup attitudes	−0.70	1.69	–	0.29	−0.01	−0.13
3	Intergroup trust	0.03	0.54		–	−0.14	−0.11
4	Outgroup perspective-taking	4.14	1.43			–	0.41
5	Relative advantage	−0.16	1.83				–

				*r*					
#	Measure	*M*	*SD*	1	2	3	4	5	6
**B** Second Survey									
1	Intergroup anxiety	3.14	1.15	−0.11	−0.05	−0.10	−0.04	−0.20	0.03
2	Intergroup attitudes	−0.41	1.33	–	0.15	−0.02	−0.16	0.22	−0.16
3	Intergroup trust	0.01	0.42		–	−0.07	−0.02	0.03	−0.02
4	Outgroup perspective-taking	4.18	1.32			–	0.36	0.11	0.05
5	Relative advantage	−0.09	1.60				–	−0.06	0.14
6	Positive contact	3.96	0.93					–	−0.09
7	Negative contact	2.08	0.89						–

				*r*						
#	Measure	*M*	*SD*	1	2	3	4	5	6	7
**C** Third Survey										
1	Intergroup anxiety	2.87	1.11	−0.14	−0.03	−0.24	0.02	−0.17	−0.17	−0.01
2	Intergroup attitudes	−0.22	1.23	–	0.31	0.03	0.00	0.05	−0.00	0.06
3	Intergroup trust	−0.01	0.36		–	0.00	−0.03	−0.00	−0.03	0.02
4	Outgroup perspective-taking	4.35	1.33			–	0.29	0.13	0.14	0.02
5	Relative advantage	−0.14	1.35				–	−0.11	−0.05	0.04
6	Contact quality	4.94	1.18					–	0.33	−0.08
7	Contact content (sum)	7.01	1.74						–	0.06
8	Contact content (difference)	−0.18	0.79							–

### Did the Intervention Improve Intergroup Relations Directly?

#### Intergroup attitudes

**Fig. 2:**
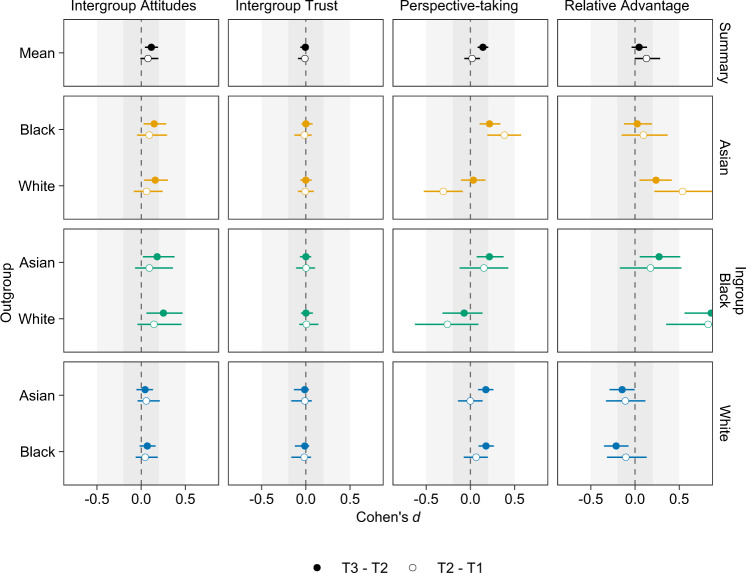
Estimated mean change in four outcome variables from T1 to T2 (before the intervention) and from T2 to T3 (during the intervention) as Cohen’s *d* effect size. When change during the intervention was greater than change before the intervention, we took this as evidence that the intervention had an effect that went beyond test–retest effects

Figure 2 shows results from the ordinal regression models for intergroup attitudes, intergroup trust, and other outcome variables. As hypothesized, participants reported, on average, more favorable intergroup attitudes after having participated in the intervention (*ΔM*_23_ = 0.21, [0.08, 0.35]; *d*_23_ = 0.11, [0.04, 0.19]). Participants did not show an improvement in intergroup attitudes during the two weeks preceding the intervention (*ΔM*_12_ = 0.14, [−0.01, 0.36]; *d*_12_ = 0.08, [−0.01, 0.19]). However, as change during the intervention did not exceed change before the intervention (*d*_23_—*d*_12_ = 0.04, [−0.12, 0.17]; Pr(*d*_23_ > *d*_12_) = 0.69),^7^ this study did not find evidence that participating in the intervention fostered more favorable intergroup attitudes.

#### Intergroup trust

For intergroup trust, this study did not find evidence for change during the intervention (*ΔM*_23_ = 0.00, [−0.04, 0.01]; *d*_23_ = −0.01, [−0.06, 0.02]), change before the intervention (*ΔM*_12_ = −0.01, [−0.05, 0.02]; *d*_12_ = −0.01, [−0.09, 0.03]), or for a difference between the two (*d*_23_—*d*_12_ = 0.00, [−0.07, 0.09]; Pr(*d*_23_ > *d*_12_) = 0.55). As Fig. 2 shows, these effects did not vary across ingroups and outgroups. Contrary to predictions, this study thus did not find evidence that participating in the intervention improved either intergroup attitudes or intergroup trust.

### Did the Intervention Improve Intergroup Relations Indirectly?

#### Intergroup anxiety

Figure 1 shows results from the ordinal regression model with intergroup anxiety as its outcome variable. As hypothesized, participants reported, on average, less intergroup anxiety after having participated in the intervention (*ΔM*_23_ = -0.27, [−0.35, −0.20]; Cohen’s *d*_23_ = −0.24, [−0.31, −0.17]). Participants, however, also showed a reduction in intergroup anxiety during the two weeks preceding the intervention (*ΔM*_12_ = −0.14, [−0.21, −0.06]; *d*_12_ = −0.12, [−0.18, −0.06]). However, as change during the intervention exceeded change before the intervention (*d*_23_—*d*_12_ = −0.12, [−0.21, −0.02]; Pr(*d*_23_ > *d*_12_) = 0.01), this study nonetheless shows that participating in the intervention reduced intergroup anxiety. As Fig. 1D shows, this study found more evidence for this effect among Black and White participants than among Asian participants.

#### Outgroup perspective-taking

Figure 2 shows results from the ordinal regression models for outgroup perspective-taking. As hypothesized, participants reported, on average, more outgroup perspective-taking after having participated in the intervention (*ΔM*_23_ = 0.16, [0.09, 0.22]; *d*_23_ = 0.14, [0.08, 0.20]). Participants did not show an increase in outgroup perspective-taking during the two weeks preceding the intervention (*ΔM*_12_ = 0.02, [−0.07, 0.12]; *d*_12_ = 0.02, [−0.07, 0.11]). As change during the intervention exceeded change before the intervention (*d*_23_—*d*_12_ = 0.12, [0.01, 0.24]; Pr(*d*_23_ > *d*_12_) = 0.98), this study found evidence that participating in the intervention increased outgroup perspective-taking. As Fig. 2 shows, this study found more evidence for change in White participants’ ratings of the Asian and Black outgroups and for change in Asian participants’ ratings of the White outgroup than for other ingroup–outgroup combinations.

### Did the Intervention Affect Perceptions of Relative (Dis-)Advantage?

#### Perceived disadvantage

As hypothesized, Asian (*ΔM*_23_ = 0.24, [0.05, 0.42]; *d*_23_ = 0.24, [0.05, 0.42]) and Black (*ΔM*_23_ = 0.87, [0.57, 1.16]; *d*_23_ = 0.86, [0.56, 1.16]) participants reported feeling less disadvantaged relative to White people after having participated in the intervention. Asian (*ΔM*_12_ = 0.54, [0.22, 0.89]; *d*_12_ = 0.54, [0.22, 0.88]) and Black (*ΔM*_12_ = 0.83, [0.36, 1.31]; *d*_12_ = 0.83, [0.35, 1.30]) participants, however, also showed a reduction in this outcome measure during the two weeks preceding the intervention. As change during the intervention did not exceed change before the intervention, this study showed that participating in the intervention did not affect Asian (*d*_23_—*d*_12_ = −0.30, [−0.74, 0.09]; Pr(*d*_23_ > *d*_12_) = 0.08) and Black (*d*_23_—*d*_12_ = 0.03, [−0.58, 0.65]; Pr(*d*_23_ > *d*_12_) = 0.54) participants’ perceptions of relative disadvantage.

#### Perceived advantage

Contrary to predictions, White participants reported feeling less advantaged relative to Asian (*ΔM*_23_ = −0.15, [−0.29, 0.00]; *d*_23_ = −0.15, [−0.29, 0.00]) and Black (*ΔM*_23_ = −0.22, [−0.35, −0.07]; *d*_23_ = −0.21, [−0.35, −0.07]) people after having participated in the intervention. White participants did not report a change in this outcome measure relative to Asian (*ΔM*_12_ = −0.11, [−0.33, 0.12]; *d*_12_ = −0.11, [−0.33, 0.12]) and Black (*ΔM*_12_ = −0.10, [−0.32, 0.13]; *d*_12_ = −0.10, [−0.32, 0.13]) people during the two weeks preceding the intervention. As change during the intervention did not exceed change before the intervention, this study showed that participating in the intervention did not reduce White participants’ perceptions of disadvantage relative to Asian (*d*_23_—*d*_12_ = −0.03, [−0.33, 0.25]; Pr(*d*_23_ > *d*_12_) = 0.40) and Black (*d*_23_—*d*_12_ = −0.11, [−0.41, 0.18]; Pr(*d*_23_ > *d*_12_) = 0.23) people.

### For Whom Did the Intervention Improve Intergroup Relations?

As this study found effects of the intervention on intergroup attitudes, intergroup trust, and perceived (dis-)advantage to not differ as a function of contact before or during the intervention, results for these outcome variables are reported in the supplemental materials.

#### Moderation by Contact Experiences Before the Intervention

**Fig. 3:**
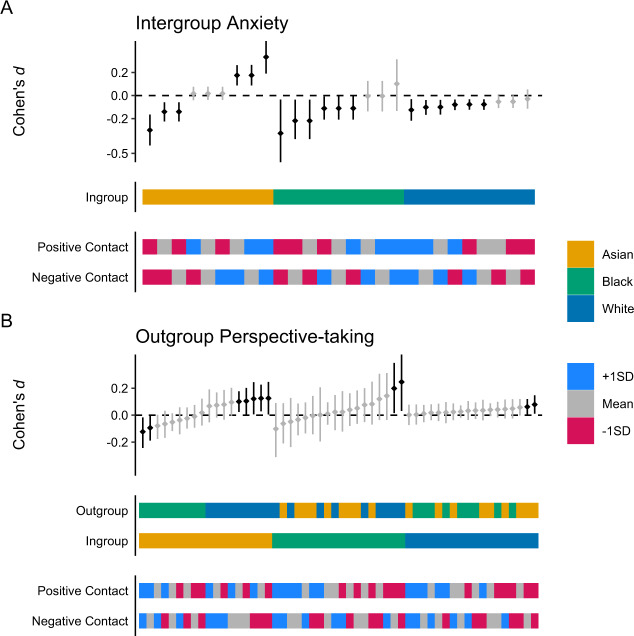
Estimated mean change during the intervention as Cohen’s *d* effect size, adjusted for change before the intervention. **A** Estimated mean change in intergroup anxiety as a function of participant ingroups and of contact experiences before the intervention. **B** Estimated mean change in outgroup perspective-taking as a function of participant ingroups and target outgroups and of contact experiences before the intervention

Figure 3 shows the estimated effect of the intervention as a function of the participants’ ingroups and their contact experiences *before* the intervention. For simplicity, this subsection only reports the estimated difference between the mean change during the intervention and the mean change before the intervention (*d*_23_—*d*_12_), rather than also report both estimated mean changes (*d*_23_, *d*_12_) separately as the previous subsection.

Figure 3A shows the estimated effect of the intervention on intergroup anxiety as a function of the participants’ ingroups and their contact experiences before the intervention. The model estimated that, if all participants had experienced less (−1*SD*) positive contact before the intervention, the intervention would have resulted in a greater reduction in intergroup anxiety across participants (*d*_23_—*d*_12_ = −0.26, [−0.37, −0.14]; Δ_−1*SD*_ = −0.13, [−0.21, −0.05]). If all participants had experienced more positive (+*SD*) contact before the intervention, the intervention would have instead resulted in no reduction in intergroup anxiety across participants (*d*_23_—*d*_12_ = 0.01, [−0.10, 0.11]; Δ_+1*SD*_ = 0.13, [0.05, 0.21]). If all participants had experienced less (−1*SD*) or more (+1*SD*) negative contact before the intervention, the effect of the intervention across participants would have remained unchanged (Δ_−1*SD*_ = −0.01 [−0.10, 0.07] and Δ_+1*SD*_ = 0.01, [−0.07, 0.10]). As Fig. 3A shows, these differences were most pronounced for Asian participants.

Figure 3B shows the estimated effect of the intervention on outgroup perspective-taking as a function of ingroups, outgroups, and the participants’ contact experiences before the intervention. The model estimated that, if all participants had experienced less (−1*SD*) positive contact before the intervention, the intervention would have resulted in a bigger increase in perspective-taking across participants (*d*_23_—*d*_12_ = 0.20, [0.06, 0.32]; Δ_−1*SD*_ = 0.09, [0.00, 0.19]). If all participants had experienced more (+1*SD*) positive contact before the intervention, the intervention would have instead resulted in no increase in perspective-taking across participants (*d*_23_—*d*_12_ = 0.00, [−0.13, 0.14]; Δ_+1*SD*_ = −0.10, [−0.20, 0.00]). If all participants had experienced less (-1*SD*) or more (+1*SD*) negative contact before the intervention, the effect of the intervention across participants would have remained unchanged (Δ_−1*SD*_ = 0.07, [−0.03, 0.17] and Δ_+1*SD*_ = −0.07, [−0.17, 0.03]).

#### Moderation by Contact Experiences During the Intervention

**Fig. 4:**
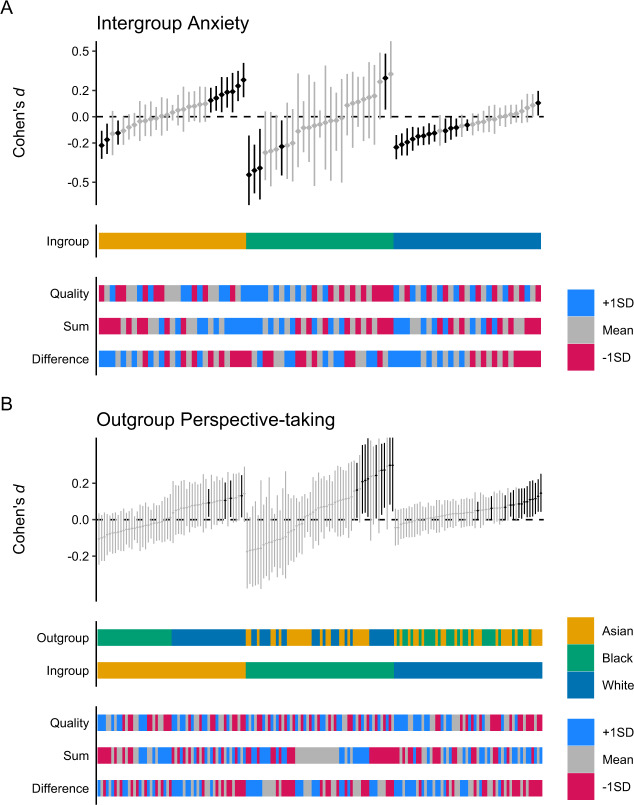
Estimated mean change during the intervention as Cohen’s *d* effect size, adjusted for change before the intervention. **A** Estimated mean change in intergroup anxiety as a function of participant ingroups and of contact experiences during the intervention. **B** Estimated mean change in outgroup perspective-taking as a function of participant ingroups and target outgroups and of contact experiences during the intervention

Figure 4 shows the estimated effect of the intervention as a function of the participants’ ingroups and their contact experiences *during* the intervention. For simplicity, this subsection again focuses on the estimated difference between the mean change during and before the intervention (*d*_23_—*d*_12_).

As Fig. 4A shows, across the three ingroups, there was an inconsistent relationship between the effect of the intervention on intergroup anxiety and the three moderator variables. The model estimated that, on average, the effect of the intervention would have remained unchanged if all participants had experienced higher (+1 *SD*) contact quality (Δ_+1*SD*_ = −0.06, [−0.16, 0.07]) or talked more (+1*SD*) about both differences and commonalities (Δ_+1*SD*_ = −0.05, [−0.16, 0.05])—or if all participants had experienced lower (−1*SD*) contact quality (Δ_−1*SD*_ = 0.05, [−0.07, 0.16]) and talked less (−1*SD*) about both differences and commonalities (Δ_−1*SD*_ = 0.05, [−0.05, 0.16]). The model estimated, however, that if all participants had talked more often (+1*SD*) about differences than about commonalities, the intervention would have resulted in a greater reduction in intergroup anxiety across participants (*d*_23_—*d*_12_ = −0.25, [−0.38, −0.13]; Δ_−1*SD*_ = −0.17, [−0.28, −0.06]). Conversely, if all participants had talked less often (−1*SD*) about differences than about commonalities, the intervention would have resulted in no reduction in intergroup anxiety across participants (*d*_23_—*d*_12_ = 0.09, [−0.04, 0.22]; Δ_−1*SD*_ = 0.17, [0.06, 0.28]).

Figure 4B shows the estimated effect of the intervention on outgroup perspective-taking as a function of ingroups, outgroups, and the participants’ contact experiences *during* the intervention. As Fig. 4B shows, the relationship between the intervention’s effect on outgroup perspective-taking and the three moderator variables was inconsistent across the six combinations of ingroups and outgroups—for different combinations, different moderators were associated with the most positive effect sizes. The model estimated that, on average, the effect of the intervention would have remained unchanged if all participants had experienced higher (+1*SD*) contact quality (Δ_+1*SD*_ = −0.08, [−0.19, 0.04]), talked more (+1*SD*) about both differences and commonalities (Δ_+1*SD*_ = 0.05, [−0.07, 0.16]), or talked (+1*SD*) more about differences than about commonalities (Δ_+1*SD*_ = −0.10, [−0.20, 0.01])—or if all participants had experienced lower (−1*SD*) contact quality (Δ_−1*SD*_ = 0.08, [−0.03, 0.19]), talked less (−1*SD*) about both differences and commonalities (Δ_−1*SD*_ = −0.06, [−0.16, 0.06]), or talked less (−1*SD*) about differences than about commonalities (Δ_−1*SD*_ = 0.10, [−0.01, 0.21]).

## Discussion

While past research has found intergroup contact to be a promising intervention to reduce prejudice and has identified adolescence as the developmental period during which intergroup contact is most effective, few studies have tested whether contact-based interventions in adolescence can be scaled up to improve intergroup relations at a large scale. The current study addressed this need for research by evaluating the effectiveness of the National Citizen Service, a large-scale contact-based intervention for 15- to 17-year-olds in the United Kingdom. Controlling for test–retest effects, this study found evidence that, on average, participating in the intervention decreased intergroup anxiety and increased outgroup perspective-taking—but not that it affected intergroup attitudes, intergroup trust, or perceptions of relative (dis-)advantage. Both effects of the intervention were small (Cohen’s *d* = 0.12). This study also found that the effects of the intervention on intergroup anxiety and outgroup perspective-taking were greater for adolescents who had experienced less positive contact before the intervention and for those who talked more about group differences in contact experiences during the intervention. These findings supported the conclusion that the National Citizen Service might not immediately improve intergroup relations, but that it has the potential to prepare adolescents for more positive intergroup contact experiences in the future.

### Theoretical Implications

Contrary to the first hypothesis, this study found no evidence that participation in the NCS *directly* improved intergroup relations. This finding contradicted previous evidence from meta-analyses of mostly correlational studies (Pettigrew and Tropp, 2006) and of intervention studies (Lemmer and Wagner, 2015) which found intergroup contact to be associated with less prejudice. This finding aligned, however, with similar research (Al Ramiah and Hewstone, 2012) and a more selective meta-analysis of intervention studies (Paluck et al., 2019) which suggested that small and non-significant effects of contact-based interventions on prejudice are not uncommon. As discussed under *Practical Implications*, this raises questions about the effectiveness of contact-based interventions that consist of short-term intergroup encounters relative to more intimate forms of intergroup contact such as cross-group friendship.

This finding also stand in contrast to Laurence’s (2020) study of the NCS which found a small but significant treatment effect on intergroup attitudes. This raises the question why Laurence (2020) found evidence for an improvement in attitudes 4–6 months after the intervention while the current study did not find evidence for an improvement immediately after the intervention.^8^ An explanation for this difference in findings might be that the intervention affects intergroup attitudes *indirectly* with effects of the intervention unfolding over time. As the current study found evidence that the intervention reduced intergroup anxiety and increased outgroup perspective-taking—and considering that these variables have been shown to increase future contact-seeking behavior (Kenworthy et al., 2016)—the present research suggests that the intervention may prepare participants for future positive contact experiences. This account corresponds to Laurence’s (2020) conclusion that positive contact experiences mediated the effects of the intervention on outgroup attitudes. Overall, this research suggests that participating in a large-scale contact-based intervention may not immediately affect intergroup attitudes but that it could lay the foundations for more positive intergroup relations to emerge in the future.

This study replicated Laurence’s (2020) finding that participation was associated with greater improvements for participants who had experienced the least positive intergroup contact prior to participation. Like Laurence (2020), this study did not find prior negative contact to moderate the effectiveness of the intervention. This study did, however, find evidence for the moderating effect of prior positive contact for two outcomes, intergroup anxiety and outgroup perspective-taking. This finding aligns with research (Page-Gould et al., 2008) which found that tasks designed to build intergroup closeness most strongly affected the anxiety and contact-seeking behavior of participants with the highest levels of pre-intervention implicit prejudice.

This study went beyond Laurence’s (2020) research as it not only examined contact *before* the intervention, but also contact *during* the intervention as a potential moderator. This study found that, at least for reducing intergroup anxiety, the intervention was more effective for participants who had talked more often about group differences than about commonalities. This finding supports the argument that intergroup contact can improve intergroup relations not by dissolving intergroup boundaries or diminishing the significance of social identities (Brewer and Miller, 1984), but by facilitating mutual intergroup differentiation (Hewstone and Brown, 1986). Another strength of the present research was that it included participants from three ingroups—two minority groups (Asian, Black) and one majority group (White)—and, unlike Laurence (2020), measured relevant outcomes in reference to all outgroups. This made it possible to differentiate between the effects of the intervention on majority–minority (Tropp and Pettigrew, 2005) as well as minority–minority (Richeson and Craig, 2011) relations. This study found more evidence for the effects of the intervention, at least for outgroup perspective-taking, on majority–minority and minority–majority relations than for the effects of the intervention on minority–minority relations (see Fig. 2). Overall, this suggests that the effectiveness of contact-based interventions depends on the participants’ contact experiences before and during the intervention as well as on their group memberships.

Contrary to past research, the current study found no evidence that participating in a contact-based intervention diminished perceptions of relative disadvantage among disadvantaged-group members (Dixon et al., 2010). This study contradicted predictions (see Reimer et al., 2017) that a contact-based intervention—in which contact situations can be expected to be structured to emphasize harmony over conflict—would provide conditions under which positive contact is likely to reduce perceptions of discrimination. Conversely, this study also contradicted hopes that the present contact-based intervention would foster awareness of their relative privilege among advantaged-group members. As such, these findings aligned with other recent research which found intergroup contact to be unrelated to perceptions of discrimination in advantaged and disadvantaged groups (Reimer et al., 2020). Again, this study does not rule out that more intimate forms of contact, such as cross-group friendship, affect support for social change, for example, by making friends from advantaged groups aware of the discrimination faced by their friends from disadvantaged groups.

### Practical Implications

These findings raise questions about the ideal *timing* of contact-based intervention. As reviewed in the introduction, adolescence is a crucial period for developing positive intergroup attitudes before they crystallize in adulthood. Based on the ‘impressionable years hypothesis’ (Krosnick and Alwin, 1989), recent longitudinal research has found that intergroup contact during adolescence might be particularly effective for fostering positive intergroup relations (Wölfer et al., 2016). In line with this research, the NCS might be considered a particularly promising intervention. As the current study found no evidence that the NCS improved interethnic attitudes, one might instead conclude that adolescence is not the best age group to target in contact-based interventions. This conclusion, however, would be premature for two reasons. First, as discussed, this study found promising evidence suggesting that the NCS decreases intergroup anxiety and increases outgroup-perspective taking. From a developmental perspective, this effect may be crucial as it occurs during a sensitive developmental period, in which social cognitions and outgroup attitudes form. Specifically, it might lay the ground and prepare adolescence for future positive spin-over effects in direct contact, as reduced intergroup anxiety, for example, was found to increase future behavioral intentions for taking up direct contact opportunities (Hutchison and Rosenthal, 2011). More broadly, being able to interact with outgroup members with confidence and being able to take their perspective are likely important life skills in diverse societies—especially when adolescents move from less diverse schools to multiethnic universities and workplaces in early adulthood. Future research should examine the long-term consequences of contact-based interventions on life outcomes in early adulthood and beyond. Second, because it did not compare the effectiveness of the intervention for other age groups, this study could not test whether the intervention would have been more effective for younger or older participants. Future research should address the relative lack of research on the effectiveness of contact-based intervention on interethnic relations among older participants.

These findings raise questions about the ideal *duration* of contact-based intervention. Pettigrew (1998) argued that “friendship potential” (that is, the extent to which a contact situation provides participants with opportunities to become friends) is an essential condition for intergroup contact to reduce prejudice. Meta-analytic evidence shows that cross-group friendship is more strongly associated with prejudice reduction than more causal forms of intergroup contact (Davies et al. 2011). One explanation for the NCS’s failure to improve intergroup attitudes might be that the program is not long enough for cross-group friendships to form that outlast the duration of the program. One implication would then be that policy makers should prioritize longer programs that go beyond short-term intergroup encounters and allow more intimate friendships to develop over time. That said, this research suggested that the NCS prepares adolescents for future intergroup contact experiences and Laurence’s (2019) research showed that the NCS, in line with this finding, leads to more interethnic ties 4–6 months later. Therefore, short-term contact-based interventions might well serve an important function in setting participants up for more positive intergroup contact in the future.

These findings raise questions about the ideal *participant* of contact-based intervention. Like Laurence (2020), the current study found participation in the NCS to be associated with greater improvements for participants who had experienced less positive contact before the intervention. While this finding suggests that contact-based interventions are most effective for participants who have had the least positive contact in the past, it should not be translated into participant recruitment practices that exclude individuals who have more experience with intergroup interactions. Although the present research cannot speak to this directly, a mixture of more and less experienced participants may be critical for program success (see Page-Gould et al. 2008). Future research on contact-based interventions should consider the composition of the participant pool with regard to their prior intergroup contact experiences. Furthermore, this study found more evidence for the effects of the intervention on majority–minority and minority–majority relations than for the effects of the intervention on minority–minority relations. This aligns with a more general impression that interventions are often designed to improve relations between minority and majority groups, but rarely focus on fostering positive minority–minority relations. Future research should consider this understudied aspect.

These findings raise questions about the ideal *content* of contact-based intervention. Extending past research, this study examined contact during the intervention as a potential moderator and found some evidence that the intervention was more effective for participants who, in intergroup interactions, talked more often about differences than about commonalities. This finding implies that contact-based interventions should follow recommendations (e.g., Ioannou et al. 2017) to include ‘intergroup’ interactions that focus on group differences instead of only focusing on commonalities. More broadly, examining contact during an intervention is crucial if one seeks to attribute its effects to its facilitation of positive intergroup contact—even though this study did not find that a common measure of contact quality was associated with the effectiveness of the intervention.

### Limitations

The current study is a rigorous evaluation of a large-scale contact-based intervention which used a pretest–posttest design to estimate its effectiveness for improving intergroup relations. As it was a government-funded and practical real-life intervention, this study was unable to randomly assign participants to an experimental and a control group. In lieu of random assignment, one needs to make stringent assumptions to infer the size and direction of the causal effect of participating in the intervention.

First, one needs to assume that the test–retest effects were consistent across time points. This study used a pretest–posttest design with a double pretest which made it possible to adjust the estimated change during the intervention (i.e., between time 2 and time 3) for the estimated change before the intervention (i.e., between time 1 and time 2). If one assumes that the size and direction of the test–retest effects were the same across both time periods—in other words, that change from time 2 to time 3 would have been of the same size and direction as change from time 1 to time 2 if participants had not participated in the intervention between time 2 and time 3—then one can interpret the estimated difference between the change during and before the intervention as an estimate of the causal effect of the intervention. It is, however, unlikely that the effect of seeing the survey for the third time would have been greater than the effect of seeing the survey for the second time and thus that this study *overestimated* the causal effect of the intervention. If one assumed that the test–retest effects were smaller between time 2 and time 3 than between time 1 and time 2, then one would expect that this study *underestimated* the causal effect of the intervention and for it to be closer to the unadjusted change during the intervention. This means that this study would still find evidence for no, or very small, average effects of the intervention on intergroup attitudes, intergroup trust, and perceived (dis-)advantage (see Fig. 2).

Second, one needs to assume that observations were missing at random. While this study achieved near-perfect retention from immediately before to after the intervention, it was only able to get one in five participants to complete the online survey two weeks before the start of the intervention. This means that it estimated the test–retest effect based on only a small subset of participants. If participants who did not complete the first survey would have, on average, reported *lower* or *higher* values on an outcome in the first survey, then this study would have, respectively, *underestimated* or *overestimated* the causal effect of the intervention. That said, this study did not find evidence that participants who completed the first survey differed from participants who did not. As such, one should consider it plausible to assume that observations were missing at random in this study.

Third, for these findings to be generalizable, one needs to assume that participants were broadly representative of future participants in the intervention. By design, participation in the intervention is voluntary. If one wants to extrapolate the present findings to years in which more adolescents participate, one needs to assume that adolescents who elected to take part do not differ in important ways from adolescents who will consider participating in the NCS in future years with more extensive recruitment. This matters because the moderator analyses suggested that the intervention was less effective for participants who had previously had more positive contact experiences. In other ways, however, readers can be confident that this sample was broadly representative of future participants since it broadly matched the ethnic and socio-economic distribution of NCS participants more broadly and of the different regions in which the intervention was offered.

All in all, this study fell short of the strict inclusion criteria of Paluck et al. (2019) meta-analysis as it included neither random assignment nor a delayed outcome measure. Still, this study improved upon many other evaluation studies with pretest–posttest designs (see Lemmer and Wagner, 2015) as (1) it recruited a large sample with near-complete retention which made it possible to estimate change during the intervention with great precision, (2) it recruited a diverse sample that included both majority-group and minority-group members which made it possible to examine majority–minority (Tropp and Pettigrew, 2005) as well as minority–minority (Richeson and Craig, 2011) relations, (3) it included a broad range of outcome measures relevant to intergroup relations, (4) it accounted for participants’ contact experiences before and during the intervention which provided a more complete picture of the role of contact in the intervention, and (5) it used a pretest–posttest design with a double pretest (Bell, 2010) which improved upon the internal validity of simpler pretest–posttest designs by controlling for test–retest effects.

## Conclusion

An evaluation a large-scale contact-based intervention found evidence for small effects of participation such that participation reduced adolescents’ anxiety about interacting with members of other ethnic groups and increased their ability to take on the perspective of members of other ethnic groups. This evaluation found that these effects were strongest for participants who had had fewer positive contact experiences before the intervention and who talked more about group differences during the intervention. Contrary to predictions, this evaluation did not find evidence that the intervention improved intergroup relations *directly* by improving intergroup attitudes and trust. Still, the current study showed that, while the intervention might not immediately improve intergroup relations, it has the potential to prepare adolescents—especially those with less positive contact experiences before the intervention—for more positive intergroup interactions in early adulthood and beyond.

## Supplementary information

Supplemental Materials

## Footnotes

1 This study considers these outcomes as a potential ‘indirect’ effect of intergroup contact on intergroup relations because—in contrast to the ‘direct’ effects reviewed before—their effects on intergroup relations are expected to unfold over time. As such, they might explain the mechanism behind Laurence’s (2019, 2020) finding that the NCS was associated with more positive contact experiences after participation. That said, this study is not able to test this mediation statistically as it did not include a later time point and as cross-sectional mediation analysis seldom yields valid results (Maxwell and Cole, 2007).

2 In the UK, “Asian” primarily refers to people of Indian, Pakistani, and Bangladeshi ethnicities who make up, respectively, 2.5%, 2.0%, and 0.8% of the population of England and Wales (Office for National Statistics, 2018).

3 A conventional cut-off for acceptable internal consistency reliability is >0.70, a condition that all measures (with two or more items) in this study exceed. What counts as acceptable test–retest reliability depends on the stability of the construct that is being measured. In this study, all outcomes are expected to change across time points and, thus, should not be expected to have perfect test–retest correlations. In these circumstances, *r* ≈ 0.50 should be considered evidence for acceptable test–retest reliability.

4 Models used *β* ∼ Student-*t* (3, 0, 1) as prior distribution for all fixed effects, *σ* ∼ Half-Student-*t*(3, 0, 3) for standard deviations of varying effects, and *Ω* ~ LKJ(2) for covariances of varying effects.

5 *M* = 1Pr(*y* = 1) + 2Pr(*y* = 2) + 3Pr(*y* = 3) + 4Pr(*y* = 4) + 5Pr(*y* = 5) = 3.05, [3.03, 3.06].

6 Models used the non-centered parameterization to model all varying effects and, where applicable, the Cholesky factorization to model covariances between varying effects.

7 Pr(*d*_23_ > *d*_12_) = 0.50 would mean that *d*_23_ < *d*_12_ and *d*_23_ > *d*_12_ have the same posterior probability, while Pr(*d*_23_ > *d*_12_) > 0.50 and Pr(*d*_23_ > *d*_12_) <0.50 would mean that there is a greater posterior probability for the intervention to, respectively, increase and decrease the relevant outcome variable.

8 It should be noted, however, that the estimated treatment effect in Laurence’s (2020) research (Cohen’s *d* = Δ*M*/*SD* = 2.58/22.06 = 0.12) falls within the uncertainty interval for the small effect found in the present research (*d* = 0.04, [−0.12, 0.17]).
