# Supplementary material for: Building Social Cohesion Through Intergroup Contact: Evaluation of a Large-Scale Intervention to Improve Intergroup Relations Among Adolescents
Source: J Youth Adolesc. 2021 Feb 18;50(6):1049–67. doi: 10.1007/s10964-021-01400-8 (PMC8116240; doi:10.1007/s10964-021-01400-8)
Supplement: Supplementary file 1 — Supplemental Materials [file 10964_2021_1400_MOESM1_ESM.docx]

# Supplemental Materials

## Analysis of Missing Data

In our analyses, we assume that whether observations were missing did not depend on participants’ responses to the outcome measures. While we achieved near-perfect retention from immediately before to after the intervention, only one in five participants completed the online survey two weeks before the start of the intervention. This means that we estimated the test–retest effect based on only a small subset of participants. If participants who did not complete the first survey would have, on average, reported *lower* or *higher* values on an outcome in the first survey, then our study would have, respectively, *underestimated* or *overestimated* the causal effect of the intervention. Here, we report additional analyses comparing participants who completed the first survey to participants who joined only for the later surveys to test how plausible our assumption about the missing data mechanism is.

Specifically, we fit a series of logistic regression models predicting whether participants completed the first survey as a function of their responses to the outcome measures in the second survey. As we estimated distinct effect sizes for each subsample, we did not need to assume that Asian, Black, and White participants were equally likely to have completed the first survey. As such, we estimated three logistic regression models, one for each subsample, that predicted participation in the first survey (1 = *yes*, 0 = *no*) as a function of participants’ intergroup anxiety ratings and their intergroup attitudes, intergroup trust, perspective-taking, and relative advantage ratings of two outgroups (e.g., Asian and Black people for White participants) in the second survey. To handle missing data in the second survey, we used multiple imputation (*m* = 100) as implemented in the *mice* package (van Buuren & Groothuis-Oudshoorn, 2011). Following recent recommendations (van Buuren, 2018; Eekhout et al., 2017), we compared each model with predictors to a null model without predictors using the *D*_3_ likelihood-ratio test for imputed datasets. Below, we report results from these tests as well as significance tests for the individual predictor variables.

For the Asian subsample, the model with predictors did not fit better than the null model without predictors (*D*_3_ = 0.77, *p* = .64); none of the regression coefficients for Asian participants’ responses to the outcome measures in the second survey were significant (all *p* > .06). For the Black subsample, the model with predictors did not fit better than the null model without predictors (*D*_3_ = 0.56, *p* = .83); none of the regression coefficients for Black participants’ responses to the outcome measures in the second survey were significant (all *p* > .09). For the White subsample, the model with predictors did not fit better than the null model without predictors (*D*_3_ = 0.40, *p* = .93); none of the regression coefficients for White participants’ responses to the outcome measures in the second survey were significant (all *p* > .19). To conclude, we found no evidence that participants’ responses to the outcome measures predicted whether they had completed the first survey or not.

## Moderator analyses

As we found the effects of the intervention on intergroup attitudes, intergroup trust, and perceived (dis-)advantage to not differ much as a function of contact before or during the intervention, we report results for these outcome variables in Figures S1 and S2.

**
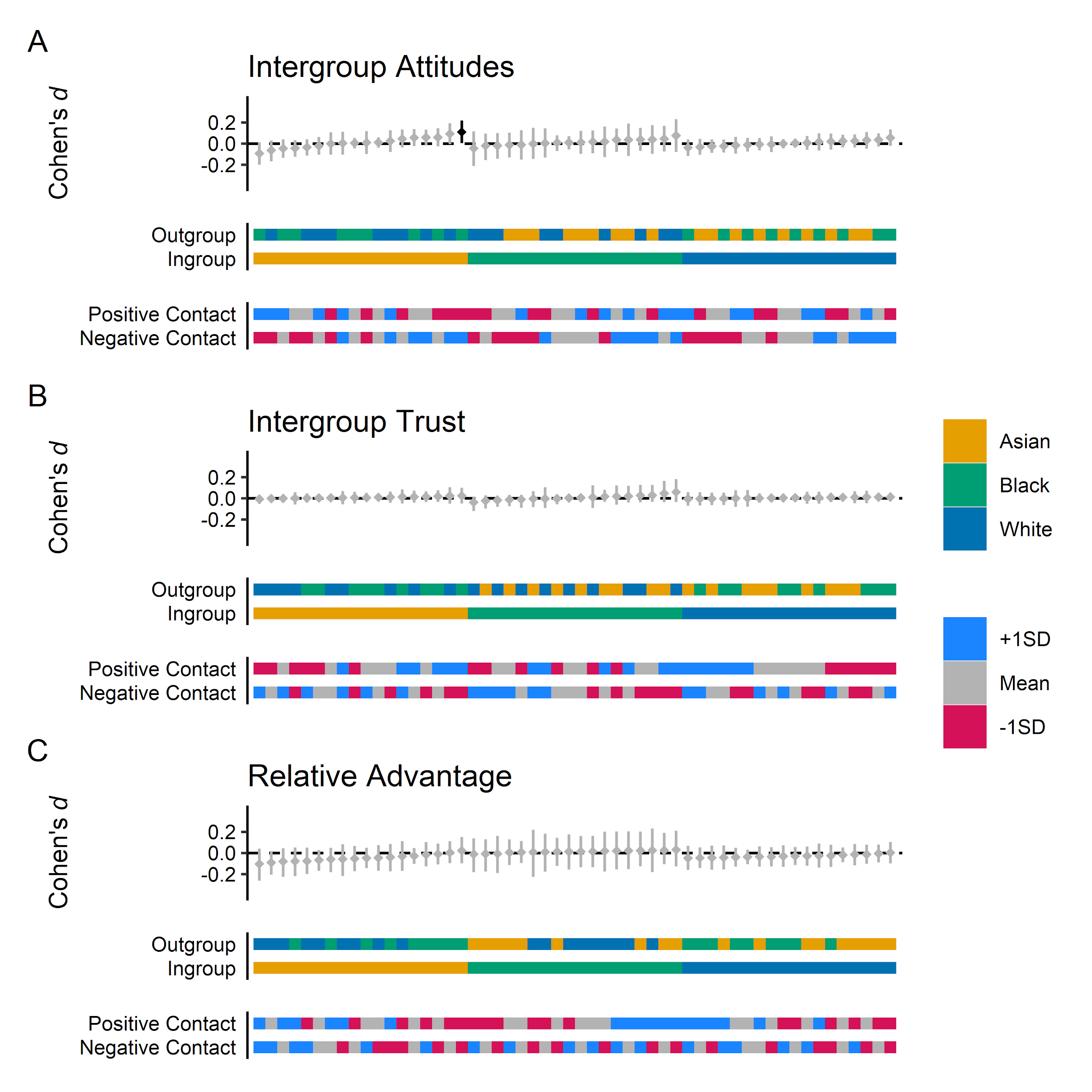
Fig. S1** Estimated mean change during the intervention as Cohen’s *d* effect size, adjusted for change before the intervention, as a function of participant ingroups and target outgroups and of contact experiences before the intervention.

*
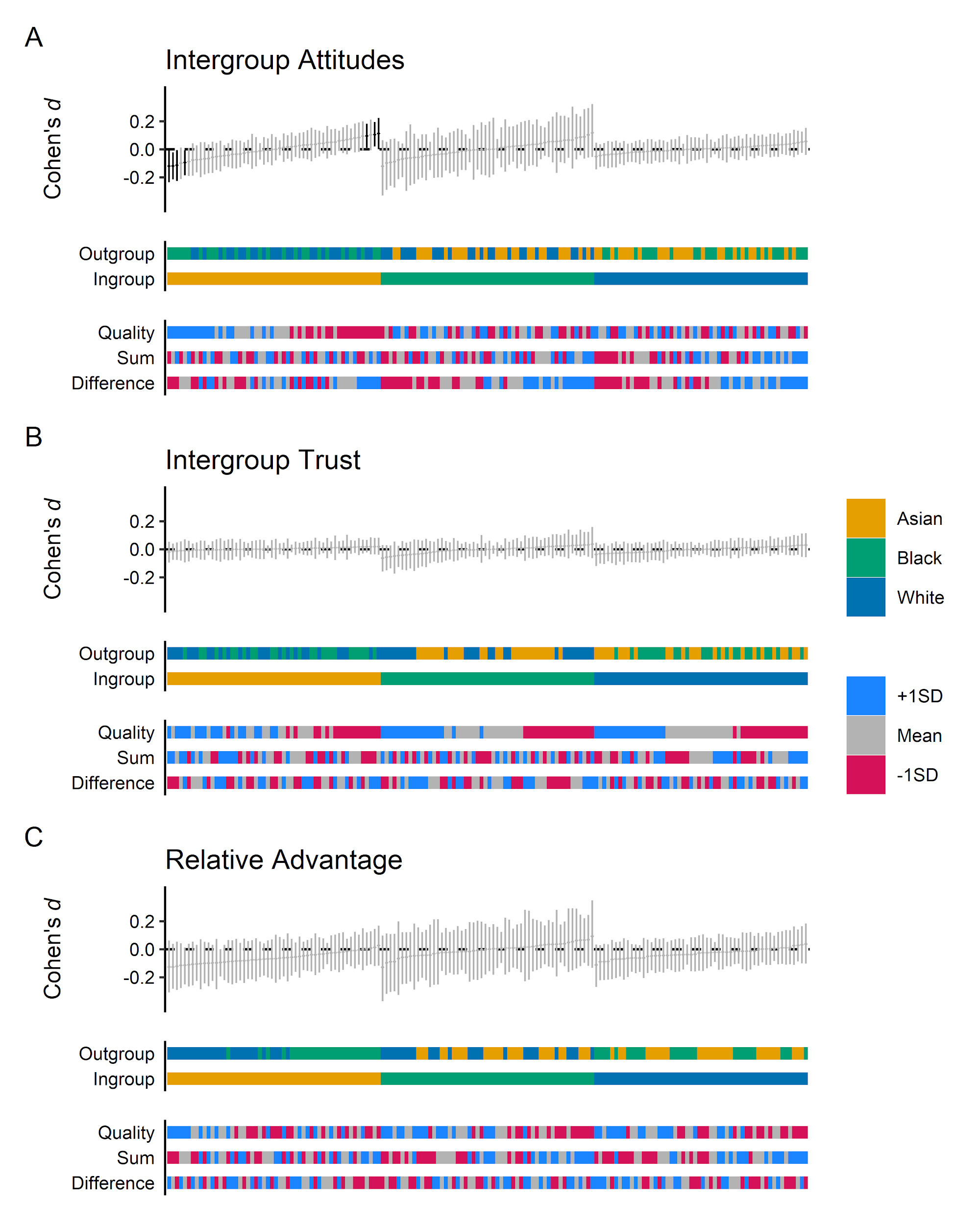
* **Fig. S2** Estimated mean change during the intervention as Cohen’s *d* effect size, adjusted for change before the intervention, as a function of participant ingroups and target outgroups and of contact experiences during the intervention.

# References

van Buuren, S., & Groothuis-Oudshoorn, K. (2011). mice: Multivariate imputation by chained equations in R. *Journal of Statistical Software*, *45*(3). https://doi.org/10.18637/jss.v045.i03

van Buuren, S. (2018). *Flexible imputation of missing data* (2^nd^ ed.). CRC Press. https://stefvanbuuren.name/fimd/

Eekhout, I., van de Wiel, M. A., & Heymans, M. W. (2017). Methods for significance testing of categorical covariates in logistic regression models after multiple imputation: Power and applicability analysis. *BMC Medical Research Methodology, 17*(1), 129. https://doi.org/10.1186/s12874-017-0404-7
